# Regulation of Neuronal Differentiation by Proteins Associated with Nuclear Bodies

**DOI:** 10.1371/journal.pone.0082871

**Published:** 2013-12-17

**Authors:** Benjamin Förthmann, Jeroen van Bergeijk, Yu-Wei Lee, Verena Lübben, Yvonne Schill, Hella Brinkmann, Andreas Ratzka, Michal K. Stachowiak, Michael Hebert, Claudia Grothe, Peter Claus

**Affiliations:** 1 Institute of Neuroanatomy, Hannover Medical School, Hannover, Germany; 2 Center for Systems Neuroscience, University of Veterinary Medicine Hannover, Hannover, Germany; 3 Department of Pathology and Anatomical Sciences, State University of New York, Buffalo, New York, United States of America; 4 Department of Biochemistry, The University of Mississippi Medical Center, Jackson, Mississippi, United States of America; University of Turin, Italy

## Abstract

Nuclear bodies are large sub-nuclear structures composed of RNA and protein molecules. The Survival of Motor Neuron (SMN) protein localizes to Cajal bodies (CBs) and nuclear gems. Diminished cellular concentration of SMN is associated with the neurodegenerative disease Spinal Muscular Atrophy (SMA). How nuclear body architecture and its structural components influence neuronal differentiation remains elusive. In this study, we analyzed the effects of SMN and two of its interaction partners in cellular models of neuronal differentiation. The nuclear 23 kDa isoform of Fibroblast Growth Factor – 2 (FGF-2^23^) is one of these interacting proteins – and was previously observed to influence nuclear bodies by destabilizing nuclear gems and mobilizing SMN from Cajal bodies (CBs). Here we demonstrate that FGF-2^23^ blocks SMN-promoted neurite outgrowth, and also show that SMN disrupts FGF-2^23^-dependent transcription. Our results indicate that FGF-2^23^ and SMN form an inactive complex that interferes with neuronal differentiation by mutually antagonizing nuclear functions. Coilin is another nuclear SMN binding partner and a marker protein for Cajal bodies (CBs). In addition, coilin is essential for CB function in maturation of small nuclear ribonucleoprotein particles (snRNPs). The role of coilin outside of Cajal bodies and its putative impacts in tissue differentiation are poorly defined. The present study shows that protein levels of nucleoplasmic coilin outside of CBs decrease during neuronal differentiation. Overexpression of coilin has an inhibitory effect on neurite outgrowth. Furthermore, we find that nucleoplasmic coilin inhibits neurite outgrowth independent of SMN binding revealing a new function for coilin in neuronal differentiation.

## Introduction

The nucleus comprises a number of subnuclear structures important for regulation of cellular functions. Several proteins are organized in endogenous and physiological aggregates which are called nuclear bodies [1], [2]. One of these structures was described as the “nucleolar accessory body” because of its closeness to the nucleolus and later named the Cajal body (CB) [3], [4]. A prominent protein that is found to be accumulated in nuclear bodies is the Survival of Motor Neuron (SMN) protein. SMN localizes to Cajal bodies (CBs) as well as to gemini of CBs (gems) [5]. Cellular decrease of SMN is responsible for pathogenesis of the neurodegenerative disease Spinal Muscular Atrophy [6]. The nuclear 23 kDa isoform of the Fibroblast Growth Factor – 2 (FGF-2^23^) exhibits an antagonistic effect on the accumulation of SMN in CBs [7]. FGF-2 is not only an extracellular factor binding to receptor tyrosine kinase receptors but also expressed as different isoforms with intracellular functions [8]. One of these isoforms, nuclear FGF-2^23^ has been shown to bind directly to SMN whereas 18 kDa FGF-2 (FGF-2^18^) does not [9], [10]. The FGF-2^23^/SMN complex negatively regulates stability of nuclear gems whereas the number of CBs remains unaffected [11]. FGF-2^23^ is imported into the nucleus with Fibroblast Growth Factor Receptor 1 (FGFR1) by importin-β [12]. The FGFR1/FGF-2^23^ complex promotes neuronal differentiation by TH-gene activation in a pathway referred to as integrative nuclear FGFR1 signaling (INFS) [13], [14], [15].

The well described marker protein for CBs is coilin [16], [17]. The major amount of coilin is not concentrated in CBs but shows a nucleoplasmic localization [18]. Possible roles for nucleoplasmic coilin are suggested in cellular stress with effects on the response to DNA damage [19], inhibition of non-homologous DNA end joining by interaction with Ku proteins [20] and accumulation at damaged centromers as a result to viral infection [21]. Coilin is also considered to play a role in U snRNP processing due to RNase activity [22]. Coilin directly binds to the SMN protein [17], [23].

The interaction between SMN and coilin is sensitively influenced by phosphorylation and methylation of coilin [24], [25], [26]. Phosphorylation of three amino acid residues of coilin (S571-572 and T573) modulates the interaction to SMN significantly. A non-phosphoryable coilin mutant (GFP-coilin AAA; S571-572A and T573A) is demonstrated to bind considerably more SMN than GFP-coilin or a phosphomimetic coilin mutant (GFP-coilin DDE; S571-572D and T573E) [26]. Essential for the association of SMN and Coilin to CBs are proteins like the zinc-finger protein ZPR1, the WRAP53 protein and INTS4, a subunit of the integrator complex [27], [28], [29].

The functional relation between architecture of the nucleus and neuronal differentiation is still elusive. In this study, we analyzed the regulatory influence of the neurotrophic and intranuclear protein FGF-2^23^ as a molecule interacting with SMN. In addition, we investigated the influence of SMN and coilin as hallmark proteins for gems and CBs, respectively, on neurite outgrowth as a paradigm for neuronal differentiation. SMN was already known to regulate neurite outgrowth [30]. On a cellular level, we found that SMN-enhanced neurite outgrowth as well as FGF-2^23^-promoted transcription was inhibited by FGF-2^23^/SMN complex formation. Both SMN and FGF-2^23^ proteins mutually antagonize the otheŕs nuclear functions. Furthermore, the SMN-interacting CB-protein coilin becomes down-regulated during neuronal differentiation, and inhibits neurite outgrowth upon overexpression. In conclusion, SMN and its interaction partners FGF-2^23^ and coilin provide a molecular link between regulation of sub-nuclear organization and neuronal differentiation.

## Materials and Methods

### Plasmids

The following plasmids have been described previously: full-length human SMN (FL-SMN) plasmid pSMN1-294-EGFP and the deletion mutant pSMN235-294-EGFP [30], Coilin-EGFP and untagged pEGFP-N1 (Clontech) [11], pEGFP-coilin, pEGFP-coilin AAA and pEGFP-coilin DDE [26] as well as pDsRed2 (Invitrogen), pFGF-2^18^-DsRed2 and pFGF-2^23^-DsRed2 [10], pCAGGS-Nurr1-3XFLAG [31] and the pCAGGS empty plasmid [32]. The reporter plasmid pNBRE3-Luc, containing three repeats of Nurr binding response elements in minimal POMC gene promoter (−34/+63) [33], is a gift from Dr. Jacques Drouin (Institut de Recherches Cliniques de Montreal). The reporter plasmid TH-Luc, containing −425/+25 bp fragment of bovine tyrosine hydroxylase (TH) promoter, has been described previously [34]. For the reporter gene assay, a β-galactosidase plasmid (pβ-gal) has been used [35]. The reference reporter plasmid (pGL4.70 [hRluc] promoterless) was purchased from Promega Corp. (Madison, WI).

### Cell types and differentiation methods

We used human neuroblastoma (NB) cells of the cell line SK-N-BE(2) [36] **.** After 24 or 72 hours hours of differentiation with 5 µM retinoic acid (RA), NB cells were analyzed morphometrically using MBF ImageJ (1.43 m) software. Similarly, we have used rat PC12 cells, which are pheochromocytoma cells of sympatho-adrenal origin, as a useful model system for neuronal differentiation [37]. Treatment of PC12 cells with nerve growth factor (NGF) drives differentiation into a sympathetic neuronal phenotype including outgrowth of neurites. Treatment with 100 ng/ml nerve growth factor (NGF) was determined after 72 hours. Cells were categorized and counted as differentiated when they contained a neurite longer than one cell diameter [30], [38]. For visualization of neurites, cells were co-transfected with a vector encoding the red fluorescent protein DsRed2 (Invitrogen). Only fluorescent cells were measured for analyses of cells after transfection. Images were acquired at room temperature with Olympus Software (CellP), using a fluorescence microscope IX70 (Olympus) equipped with an objective LC Plan FI (40x/numerical aperture 0.40) and a cooled CCD camera XM10 (Olympus) or using a BX60 microscope (Olympus) with an oil immersion objective U Plan FI (100x/numeric aperture 1.30) and the same camera.

### Colocalization analysis

The nuclei of randomly selected cells (24h –RA, n = 18; 24h +RA, n = 13; 72h +RA, n = 20; pooled from 2 independent experiments) were analyzed by colocalization analysis using MBF ImageJ (1.43 m) with Intensity Correlation Analysis plug-in [31], [39], [40]. Mandeŕs Overlap Coefficient (R) was calculated by the ratio of the intersecting volume to the total object volume with a total pixel intensity >0, ranging from zero to one, representing low to high colocalization.

### Immunocytochemistry, fluorescence microscopy and western blot

Immunocytochemistry and fluorescence microscopy were performed as described previously [7]. Antibodies used were anti-coilin (H-300, rabbit IgG, Santa Cruz), anti-SMN (mouse IgG, BD Bioscience), anti-α-tubulin (mouse IgG, Santa Cruz) and secondary antibodies Alexa Fluor® 488 goat anti-mouse IgG, Alexa Fluor® 555 goat anti-mouse IgG and Alexa Fluor® 555 goat anti-rabbit IgG (Invitrogen) or anti-mouse peroxidase-linked (sheep, Amersham) and anti-rabbit peroxidase-linked (goat IgG, Jackson ImmunoResearch).

Proteins were extracted by sonification in modified RIPA-buffer [50 mM Tris/HCl, pH 7.5, 150 mM NaCl, 2 mM dithiothreitol (DTT), 1 mM EDTA, 1% (v/v) NP-40, 0.25% (w/v) sodium desoxycholate, 1 x Complete protease inhibitor (Roche)] followed by incubation for 30 min on ice and subsequent centrifugation. SDS-PAGE and Western blot were carried out as previously described [7].

### Reporter gene assays

Transfections of neuroblastoma cells were performed with Lipofectamine 2000 (Invitrogen) at ∼60% confluency in 24-well plates. The cells were harvested 36 h after transfection. Each sample/well for transfection contained a total of 1 µg of DNA, including 0.3 µg of reporter plasmid, 0.3 µg of reference reporter plasmid (pGL4.70 [hRluc] promoterless), 0.3 µg of FGF-2 isoforms plasmids (or pcDNA3.1 as negative control), 0.3 µg of pSMN1-294 or the mutant pSMN235-294 (or a vector for β-galactosidase pβ-gal as a negative control), 10 to 100 ng of pCAGGS-Nurr1 expression vector (pNurr1) and pCAGGS empty vector to make up the total amount. Dual luciferase assays were performed with the dual luciferase reporter assay system (Promega Corp., Madison, WI). All reagents were prepared as described by the manufacturer. The 5x passive lysis buffer was supplied by the manufacturer and used for cell lysis. After two washing steps, NB cells were directly transferred into another reaction tube with 100 µl of 1x passive lysis buffer. After lysis for 15 to 20 min, a 20 µl aliquot was used for luminescence measurements with BioTek Plate Reader. The following steps were performed for luminescence measurements: 100 µl of the firefly luciferase reagent (LARII) was added to the test sample, with a 10 sec equilibration time and measurement of luminescence with a 2 sec integration time, followed by addition of 100 µl of the Renilla luciferase reagent and firefly quenching (Stop & Glo) with the same equilibration time and measurement of luminescence. The data is represented as the mean ± SEM of the ratio of firefly to Renilla luciferase activity (Fluc/Rluc) for 2 to 4 experiments each performed in quadruplicate.

## Results

### Interaction of SMN with FGF-2^23^ negatively regulates neurite outgrowth

**Figure 1:**
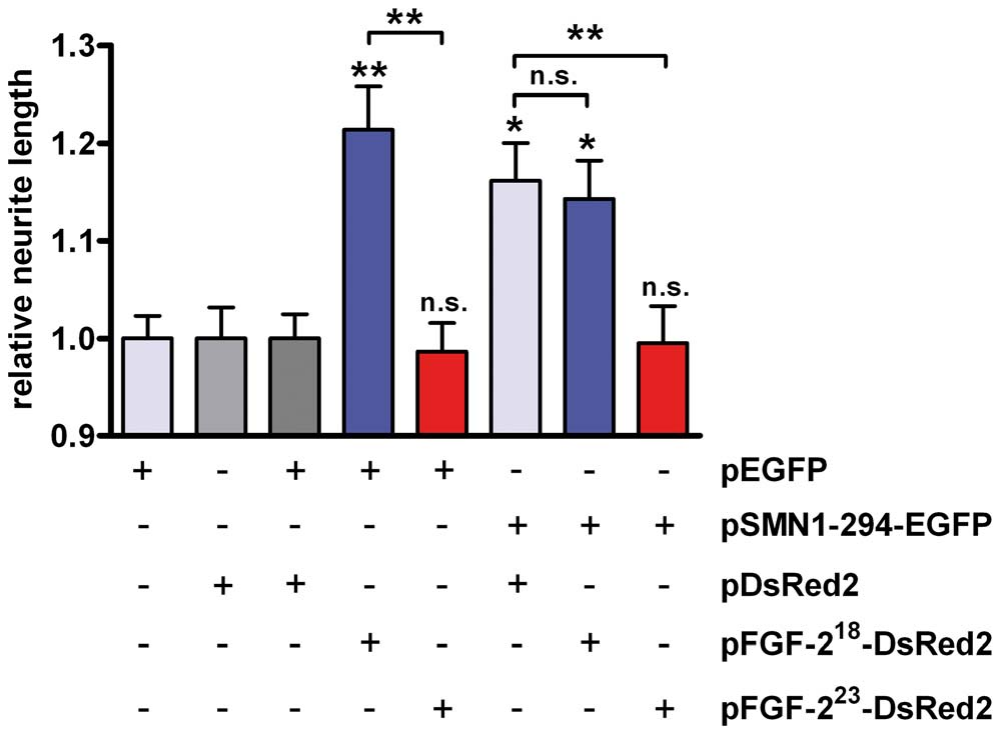
Interaction of SMN with FGF-2^23^ negatively regulates neurite outgrowth. PC12 cells were transfected with pSMN-EGFP or pEGFP control vector, respectively. Cells were additionally transfected with pFGF-2^18^-DsRed2 (SMN non-interacting control), pFGF-2^23^-DsRed2 (SMN-interacting) or pDsRed2 control (grey, transfections with control plasmids; blue, FGF-2^18^ and, red, FGF-2^23^ transfections). After incubation with nerve growth factor (NGF) in differentiation medium for three days, neurite lengths were measured and the relative changes of lengths analyzed. For statistical evaluations, average relative neurite lengths of n>100 cells were compared. Levels of significance on top of each bar represent comparison to pEGFP/pDsRed2 controls; means ± SEM; n.s., non-significant; *, p<0.05; **, p<0.01; Dunn’s test for comparison of multiple groups.

How does FGF-2^23^ affect the function of SMN in neuronal differentiation? To address this question we analyzed the effects of FGF-2^23^ and SMN in a neurite outgrowth model using PC12 cells. We coexpressed FGF-2^23^ with full-length SMN (SMN1-294) as fusion constructs with the fluorescent proteins EGFP and DsRed2, respectively. After transfection and three days of differentiation with NGF the lengths of neurites were measured. Fluorescently tagged FGF-2^23^ associated with chromatin and was found in the nucleoplasm. FGF-2^18^ localized to nucleoli and nucleoplasm. For both FGF-2 isoforms no peculiar co-localization with SMN was found in nuclear bodies (Fig. S1). Therefore, the interaction likely occurred in the nucleoplasm. The data are in agreement with the previously described localization patterns of both isoforms in Schwann cells [10] We have previously shown that the intracellular 18 kDa FGF-2 isoform (FGF-2^18^) is able to induce neurite outgrowth and neuronal differentiation of PC12 cells even in the absence of extracellular NGF [41], [42] in comparison to FGF-2^23^. In addition, FGF-2^23^ has been shown to directly interact with SMN. In contrast, FGF-2^18^ does not interact with SMN and is therefore a stringent negative control to test effects on neurite outgrowth via SMN [9]. As expected, NGF-treated PC12 cells expressing FGF-2^18^ displayed significantly longer neurites than cells expressing a pDsRed2 control plasmid or FGF-2^23^ in control conditions with pEGFP coexpression (Fig. 1). No significant difference was detected between pFGF-2^23^-DsRed2 and control pDsRed2 transfected cells. Full-length SMN (1-294) positively regulated neurite outgrowth in agreement with previous data [30], [38], [43], [44]. Interestingly, coexpression of FGF-2^23^ led to a suppression of the SMN neurite growth promoting effect (Fig. 1). Lengths of neurites were significantly reduced compared to SMN expressing cells. As a control, the SMN non-binding FGF-2^18^-isoform [9] did not show this regulatory action. Therefore, the data argue for a negative regulatory role of the SMN/FGF-2^23^ complex for SMN promoted neurite outgrowth.

### SMN inhibits activation of transcription by FGF-2^23^

**Figure 2:**
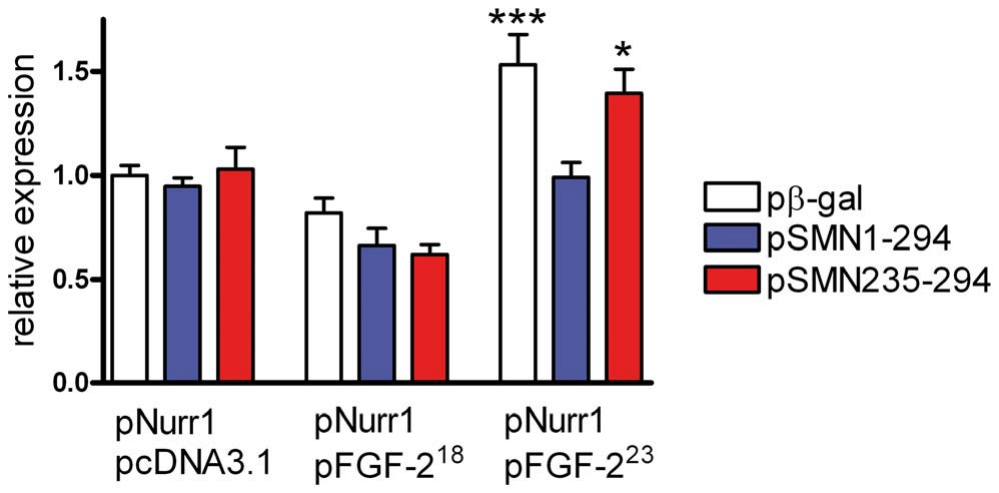
SMN inhibits FGF-2^23^ activation of Nurr1-dependent transcription. In this reporter-gene assay, the effects of FGF-2^23^, Nurr1 and SMN on transcription driven from the Nurr1 monomer binding responsive element (NBRE) were assessed by transfection of neuroblastoma cells NB. For this purpose, expression vectors for each protein and the NBRE-Luc reporter were used. Empty vector pcDNA3.1 was employed as a FGF-2 expression negative control and pβ-gal as a negative control for SMN constructs. Full-length-SMN (SMN1-294) inhibits transcriptional activation mediated by FGF-2^23^, whereas coexpression of the SMN mutant protein SMN235-294 (without the N-terminal FGF-2^23^-binding sequence and comprising amino acid residues 235-294) did not exhibit an inhibitory effect. Data represent the mean ± SEM of the ratio of firefly to *Renilla* luciferase activity (Fluc/Rluc) for n = 3 experiments, each performed in quadruplicate. Results were analyzed using one-way ANOVA followed by Tukeýs posthoc test (means ± SEM; ***, p<0.001; *, p<0.05; compared with pcDNA3.1/pNurr1.

Due to the inhibiting effect of FGF-223 on SMN-promoted neurite outgrowth of PC12 cells, we were also interested in elucidating the impact of SMN on FGF-223 functions. In the integrative nuclear FGFR1 signaling (INFS) pathway [13], [14], FGF-223 and its binding partner nuclear FGFR1 act as transcriptional coactivators which alone have no effects on gene expression but augment functions of diverse sequence-specific transcription factors including CREB, AP1 and NFκB [45]. FGFR1 binds the common coactivator CREB-binding protein (CBP) [45]. Recently, we could show that FGFR1 acts as a coactivator for the Nurr1 transcription factor [31], which controls developmentally important genes in neuronal differentiation [31], [33]. We used this system as a model to test transcriptional activity augmented by FGF-2 in the presence of SMN (Fig. 2). SK-N-BE(2) neuroblastoma (NB) cells were transfected with the luciferase reporter gene controlled by a common Nurr1 monomer binding responsive element (NBRE). This element has an important regulatory role in tyrosine hydroxylase (TH)-dependent neuronal differentiation [46]. Here, the NBRE induced the expression of luciferase activity in the presence of cotransfected Nurr1 (Fig. 2). No such expression was detected in the absence of Nurr1 (data not shown). In comparison to the negative control FGF-218, FGF-223 is able to coactivate Nurr1-mediated transcription, which is abolished by cotransfected full length SMN (pSMN1-294). In contrast, the SMN mutant (pSMN235-294) with truncated N-terminal FGF-223 binding region [9] had no significant effect on the FGF-223 coactivation. Therefore, SMN-binding to FGF-223 displays an inhibitory function in the regulation of transcriptional activity at Nurr1-regulated sequences as a molecular model in the context of neuronal differentiation.

### Regulation of the SMN-binding partner coilin and Cajal bodies in neuronal differentiation

**Figure 3:**
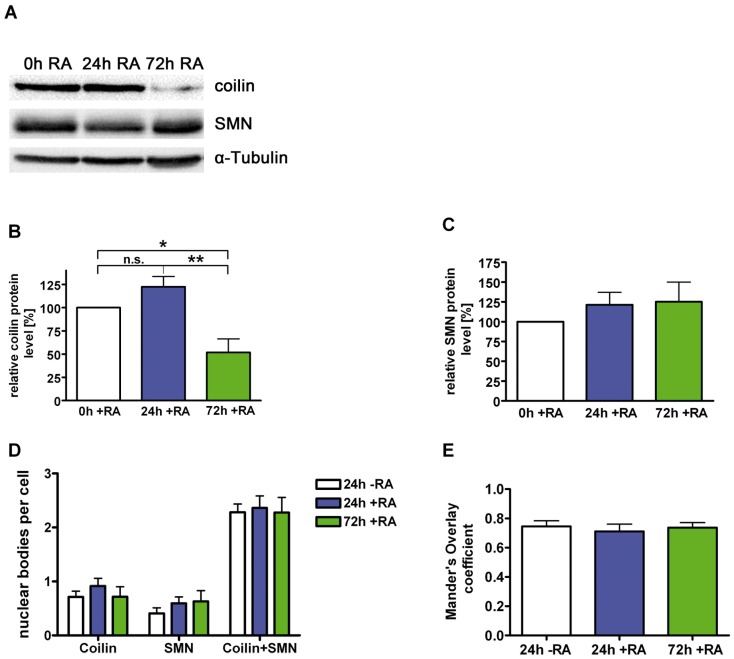
Regulation of the SMN-interacting protein coilin. Neuroblastoma SK-N-BE(2) cells were differentiated for different periods with retinoic acid (5 µM), lysed by sonification in modified RIPA-buffer [7] and analyzed by Western blot for coilin, SMN and α-Tubulin (A). (B) Endogenous coilin protein levels were decreased significantly to 52% after 72h of differentiation, (C) while SMN protein levels were increased non-significantly after the same time of differentiation. (D) Cells were treated with RA and stained with anti-SMN and anti-coilin antibodies. Coilin-positive (SMN-negative Cajal bodies), SMN-positive (nuclear gems) as well as coilin- and SMN-positive dots (SMN-positive CBs) in the nucleus of differentiated NB cells were counted and compared with a non-differentiated control. No significant changes of absolute numbers of these nuclear bodies were detected. (E) Intensity correlation analyses of coilin and SMN in the nucleus of differentiated cells were performed and compared to a non-differentiated control. No change in colocalization of coilin and SMN was detected (B, C: n = 7, means ± SEM; *, p<0.05; **, p<0.01; unpaired, two tailed t-test; D: control, n = 18; 24h RA, n = 13; 72h RA, n = 20; E: control, n = 20; 24h RA, n = 15; 72h RA, n = 22).

SMN and its binding partner coilin in Cajal bodies are both developmentally regulated during neuronal differentiation of PC12 cells. While SMN becomes upregulated, expression of coilin is down-regulated after treatment of cells with NGF [30]. To further elucidate the role of coilin in neurons, we have analyzed coilin expression and nuclear body profiles in the retinoic acid (RA) paradigm of neuronal differentiation. RA enters the cell, translocates to the nucleus and binds directly to its co-transcription factors retinoic acid receptor (RAR) and retinoid X receptor (RXR) thereby regulating RA-dependent genes [47]. After lysis of RA- differentiated NB cells, expression of coilin was analyzed on western blots (Fig. 3A, B). 24 hours later, a slight non-significant increase of the coilin protein level was detected. After 72 hours of differentiation, the coilin level was decreased to 52% (Fig. 3A, B). SMN levels were not altered significantly during this period but displayed a tendency for upregulation (Fig. 3A, C).

Human coilin is able to effectively form CBs at low levels in HeLa cells, but becomes diffusely nucleoplasmic at higher levels of expression [48]. To analyze whether reduced levels affect the coilin concentration in nuclear bodies, we have counted the number of coilin-positive (Cajal bodies, without SMN), SMN positive (nuclear gems, coilin-negative) as well as coilin- and SMN- positive dots (CBs with SMN) in nuclei of differentiated cells (Fig. 3D). No significant change of the total numbers was found (Fig. 3D) suggesting that only the diffusely distributed coilin in the nucleoplasm was reduced in NB cells. Quantitative colocalization analyses were performed to explore variances in the coilin and SMN interaction in the nucleus of differentiated cells [31], [39], [40]. No significant changes of Mandeŕs Overlap coefficient were found in nuclear bodies (Fig. 3E) indicating that coilin/SMN binding was not changed. Therefore, down-regulation of nucleoplasmic coilin in differentiating neuroblastoma cells putatively has no effect on nuclear body formation or SMN binding.

### Antagonistic roles of coilin and SMN in neuronal differentiation

**Figure 4:**
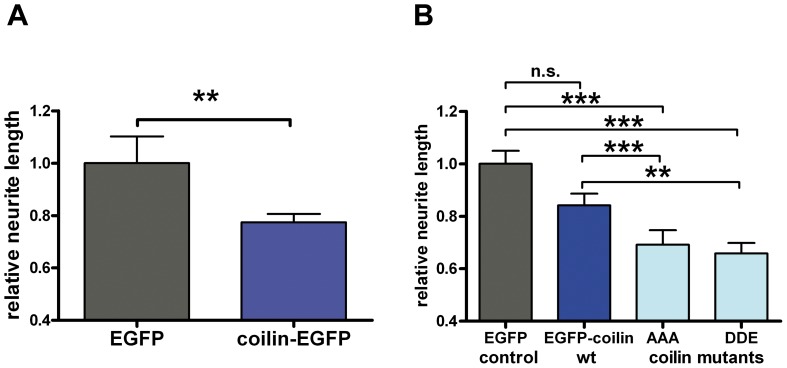
Expression of coilin decreases neurite outgrowth. (A) PC12 cells were transfected with pEGFP or pCoilin-EGFP. After incubation in differentiation medium with nerve growth factor (NGF) for three days, neurite lengths were measured and their relative changes analyzed. (B) N-terminal EGFP-tagged wild-type and mutant coilin-constructs decreased the length of neurites significantly in the same experiment. For statistical evaluation, averaged relative neurite lengths of n>100 cells were compared, pooled from three individual experiments (means ± SEM; n.s.; A: **, p<0.01; Mann-Whitney test; B: n.s., non-significant; *, p<0.05; **, p<0.01; ***, p<0.001; Dunn’s test for comparison of multiple groups).

Since the SMN binding partner FGF-2^23^ negatively regulates SMN-dependent neurite outgrowth (Fig. 1), expression of the SMN interaction partner coilin may also have a negative regulating effect on neurite outgrowth. To analyze this, we measured neurite lengths of PC12 cells expressing coilin-EGFP (Fig. 4A). Wild-type coilin expression had a significantly negative impact on neurite lengths compared to EGFP controls (Fig. 4A). The C-terminal fusion protein coilin-EGFP displays abnormal localization patterns in the cell nucleus [48], [49]. To exclude that the detected effects were due to altered coilin-EGFP functions by its C-terminal fusion with EGFP, we repeated the measurements with a coilin construct tagged at the N-terminus (EGFP-coilin) (Fig. 4B). Decrease of neurite lengths was observed independent from orientation of the EGFP-tag (Fig. 4A, B).

Furthermore, we addressed the question whether coilin binding to SMN altered its function in neuronal differentiation. Therefore, we used a non-phosphoryable coilin-mutant (EGFP-coilin AAA) and a phosphomimetic coilin-mutant (EGFP-coilin DDE). In these coilin triple-mutations of the SMN-binding sequence, the amino acid residues SST (571-573) were changed to AAA or DDE, respectively [26]. Compared to coilin AAA, coilin DDE displays reduced SMN-binding affinity [26]. EGFP-coilin as well as coilin mutans were not found concentrated in Cajal bodies in most of the cells but diffuse nucleoplasmic (Fig. S2), as it was already shown for human coilin in murine embryonic fibroblasts [48]. After differentiation, PC12 cells transfected with pEGFP-coilin AAA and pEGFP-coilin DDE, respectively, both exhibited significantly decreased neurite outgrowth compared to EGFP control and EGFP-coilin wildtype expressing cells (Fig. 4B). This indicates that the negative regulatory effect of wild-type-coilin is independent of its phosphorylation state and of SMN-binding since both mutants decrease neurite outgrowth similarly. The differences between the effects of mutant- and wild-type-coilin could be due to allosteric effects rather than to be regulated by phosphorylation. In conclusion, promotion of neurite growth by SMN and outgrowth-suppression by coilin act antagonistically. Moreover, the negative regulatory role of coilin appears to be SMN-independent.

## Discussion

In this study, we focused on the role of three nuclear proteins, the Cajal body components coilin and SMN and the SMN-interaction partner FGF-2^23^. We have discovered multiple antagonistic effects: while coilin shows an SMN-independent inhibitory effect on neurite outgrowth of PC12 cells, SMN promotes neuronal differentiation [30] . This function of SMN is mutually antagonized by nuclear FGF-2^23^. On a transcriptional level, SMN inhibits activity of a FGF-2^23^-responsive gene.

As shown in this study, FGF-2^23^ inhibited SMN-mediated neuronal differentiation. This is in agreement with previous data demonstrating stabilization of the endocrine phenotype of PC12 cells by upregulating of nuclear FGF-2^23^ whereas intracellular FGF-2^18^ shifts cells towards neuronal differentiation [41]. *In vivo*, FGF-2^23^ protein becomes selectively and strongly upregulated about 11-fold compared to 4-fold of FGF-2^18^ during regenerative conditions in spinal ganglia and after peripheral nerve lesion [50]. These results demonstrate a specific physiological function of FGF-2^23^-upregulation under these conditions. In line with our data, in the presence of FGF-2 peripheral nerve regeneration is reduced whereas FGF-2 knockout mice show faster nerve recovery [51], [52]. Besides a crucial role in the early phase of recovery, FGF-2^23^ putatively restricts nerve regeneration in a later phase.

The mutual interaction between FGF-2^23^ and SMN represses neuronal differentiation as shown here by (a) inhibiting of Nurr1-dependent transcription relevant e.g. for regulation of the tyrosine hydroxylase gene (FGF-2^23^-promoted) and (b) negative regulation of neurite outgrowth (SMN-promoted). This is of physiological relevance since FGF-2^23^ becomes selectively upregulated under regenerative conditions [66]. A nuclear function of SMN has still not been defined. However, we demonstrate that SMN can be considered as an inhibitory nuclear factor antagonizing the effects of FGF-2^23^. The ability of FGF-2^23^ to augment transcription by the orphan nuclear receptor Nurr1 is effectively regulated (turned off) by increasing the expression of SMN. This effect is not observed with truncated SMN, which lacks the FGF-2^23^ binding domain, suggesting that SMN/FGF-2^23^ complex formation blocks its coactivator function. Through these mechanisms, SMN may control functions of diverse FGF-2^23^ responsive genes and thereby influence cell development.

The functional connection between nuclear body structure and neuronal differentiation is probably more than just a correlation. The architecture of the nucleus changes during neuronal differentiation [53]. During embryonic development, coilin and SMN localize to different nuclear body structures [54], but transcription of neuroblastoma SH-SY5Y cells with retinoic-acid increases their colocalization [55]. Moreover, CBs move in the vicinity of nucleoli upon stimulation of PC12 cells with nerve growth factor [56]. Coilin dynamics are preceded by increased neuronal transcription in rat hippocampus neurons [57]. The total number of CBs correlates with the size of a neuron [58]. Importantly, increased recruitment of SMN from the nuclear pool to CBs is observed during neuritogenesis [59]. Furthermore, overexpression of SMN promotes neurite outgrowth [30], [44]. Together, these findings indicate that nuclear coilin and SMN exhibit specific roles in neuronal development. In our study, we show decreased coilin levels during differentiation of NB cells induced by retinoic acid in accordance with previous data observed in NGF-treated PC12 cells [30]. However, developmental down-regulation of coilin did not affect the total number of CBs. Therefore, the level of coilin available in CBs is still high enough to maintain CBs. This explains why SMN can still become recruited to CBs in the process of neuronal differentiation [59]. Overexpression of N- and C-terminal tagged coilin-constructs both decreased neurite lengths of PC12 cells. It has been previously shown the orientation of fusion tags with coilin influences coilin localization. Physiological trafficking between cytoplasm and the nucleus might be impaired in EGFP-coilin constructs [49]. Therefore, wildtype coilin and mutants were tested with EGFP at the N-terminal position in this study. Although many functions of coilin are linked to SMN [17], [60], [61], its activity during differentiation of NB cells appears to be independent of SMN. Mutants of coilin that bind SMN with different affinities (coilin AAA and coilin DDE) show a similar effect on neurite outgrowth like wild-type-coilin, arguing against a SMN-dependent role. The antagonistic effects of the SMN binding partners coilin and FGF-2^23^ on SMN functions are probably not functionally connected. While coilin is down-regulated during differentiation and decreases neurite outgrowth, even an upregulation of FGF-2^23^ has not a negative effect on neurite outgrowth compared to control vector. Only the neurite promoting effect caused by SMN upregulation is affected by FGF-2^23^.

Coilin binds single- and double-stranded DNA, interacts with snRNAs and comprises DNA-dependent and specific RNase activity [22], [62]. This argues for a model in which non-CB associated diffuse coilin could interfere with neuronal differentiation by degradation of specific RNAs. SnRNP association to coilin depends on the function of SMN in transporting snRNPs to CBs [61]. In contrast, RNase activity has been shown for purified coilin [22] demonstrating a function independent of SMN-binding. This coilin-activity is in line with the influence of coilin on neuronal differentiation, which appears to be independent of SMN.

Coilin plays an important role in development. Zebrafish embryos, depleted of coilin, display reduced cell proliferation and a developmental arrest due to deficits in mRNA maturation and snRNP biogenesis [63]. Residual Cajal bodies, void of coilin, are not able to restore their function, but injection of mature human snRNPs are sufficient to bypass the defect [64]. Interestingly, neuronal differentiation in Zebrafish is associated with a decreased CB number. Up to 30 CBs are detected in embryonic cell nuclei whereas neurons display a steady state level of two Cajal bodies [65]. No data of the total coilin protein amounts before and after neuronal differentiation are available, so it is impossible to compare the outcome of this model directly with our analysis. However, we find an identical steady state of two Cajal bodies per nucleus in neuroblastoma cells.

Interestingly, the employed cellular models for neuronal differentiation differ in induction and signaling pathways, but show similarities in the negative regulating role of coilin. Retinoic acid crosses the cell membrane and binds to diverse members of the RA receptor family in the cell. RAR/RXR heterodimers bind RA response elements (RARE), thereby influencing gene expression in a complex manner. This results in the induction of differentiation of NB cells [47], [66]. In contrast, NGF binds cell surface receptors (p75NTR, TrkA), activates signaling cascades including Ras and extracellular signal-regulated kinase (ERK), resulting in gene transcription in PC12 cells [67]. Endogenous coilin is down-regulated in both models and overexpression of coilin decreases the efficiency of neurite outgrowth in PC12 cells. These findings strongly suggest a novel function for the CB marker coilin in the interference of neuronal differentiation.

## Supporting Information

Figure S1 **FGF-2 isoforms do not alter the nuclear SMN distribution in PC12 cells.** PC12 cells were transfected with pFGF-2^18^-DsRed2 (A) or pFGF-2^23^-DsRed2 (B), differentiated with nerve growth factor (NGF) for 72h and immunostained for SMN. The major amount of FGF-2^18^-DsRed2 (A) localizes to nucleoli and nucleoplasm. FGF-2^23^-DsRed2 (B) is chromatin associated and localizes to the nucleoplasm. SMN shows a nuclear localization in nuclear bodies (arrows). Scale bar, 5 µm.
(TIF)

Figure S2 **Coilin constructs are similarly distributed in the nucleus of PC12 cells.** After transfection with human pEGFP-coilin (A), pEGFP-coilinAAA (B) or pEGFP-coilinDDE (C), respectively, and NGF differentiation for 72 hours, PC12 cells were immunostained for SMN. Overexpressed coilin was not found to be accumulated in Cajal bodies in most of the cells. Most SMN positive nuclear bodies were negative for human coilin, too. Scale bar, 5 µm.
(TIF)
