# Supplementary figures and images for: Regulation of Neuronal Differentiation by Proteins Associated with Nuclear Bodies
Source: PLoS One. 2013 Dec 17;8(12):e82871. doi: 10.1371/journal.pone.0082871 (PMC3866168; doi:10.1371/journal.pone.0082871)

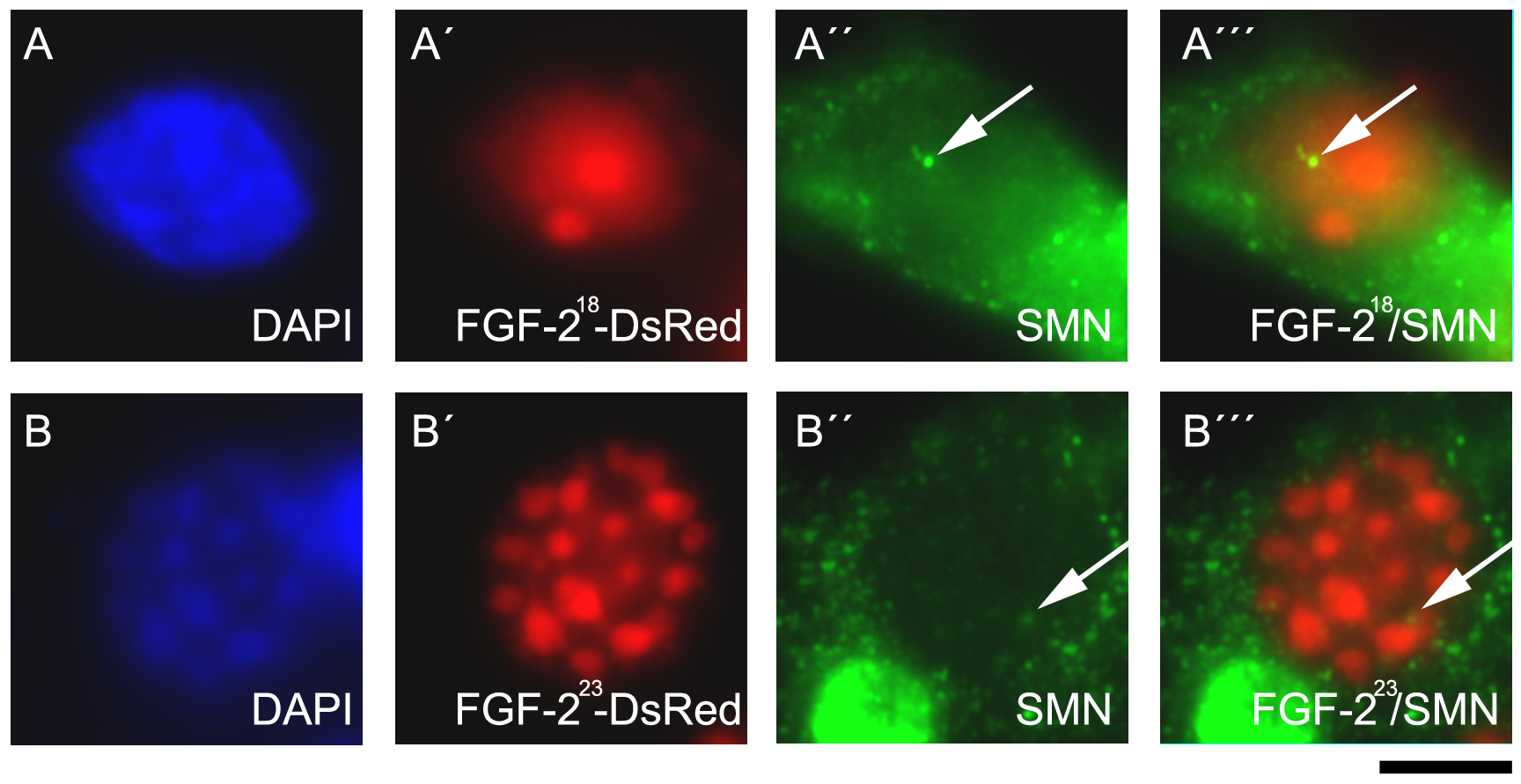

Supplement: Figure S1 — FGF-2 isoforms do not alter the nuclear SMN distribution in PC12 cells. PC12 cells were transfected with pFGF-218-DsRed2 (A) or pFGF-223-DsRed2 (B), differentiated with nerve growth factor (NGF) for 72h and immunostained for SMN. The major amount of FGF-218-DsRed2 (A) localizes to nucleoli and nucleoplasm. FGF-223-DsRed2 (B) is chromatin associated and localizes to the nucleoplasm. SMN shows a nuclear localization in nuclear bodies (arrows). Scale bar, 5 µm. (TIF) [file pone.0082871.s001.tif]

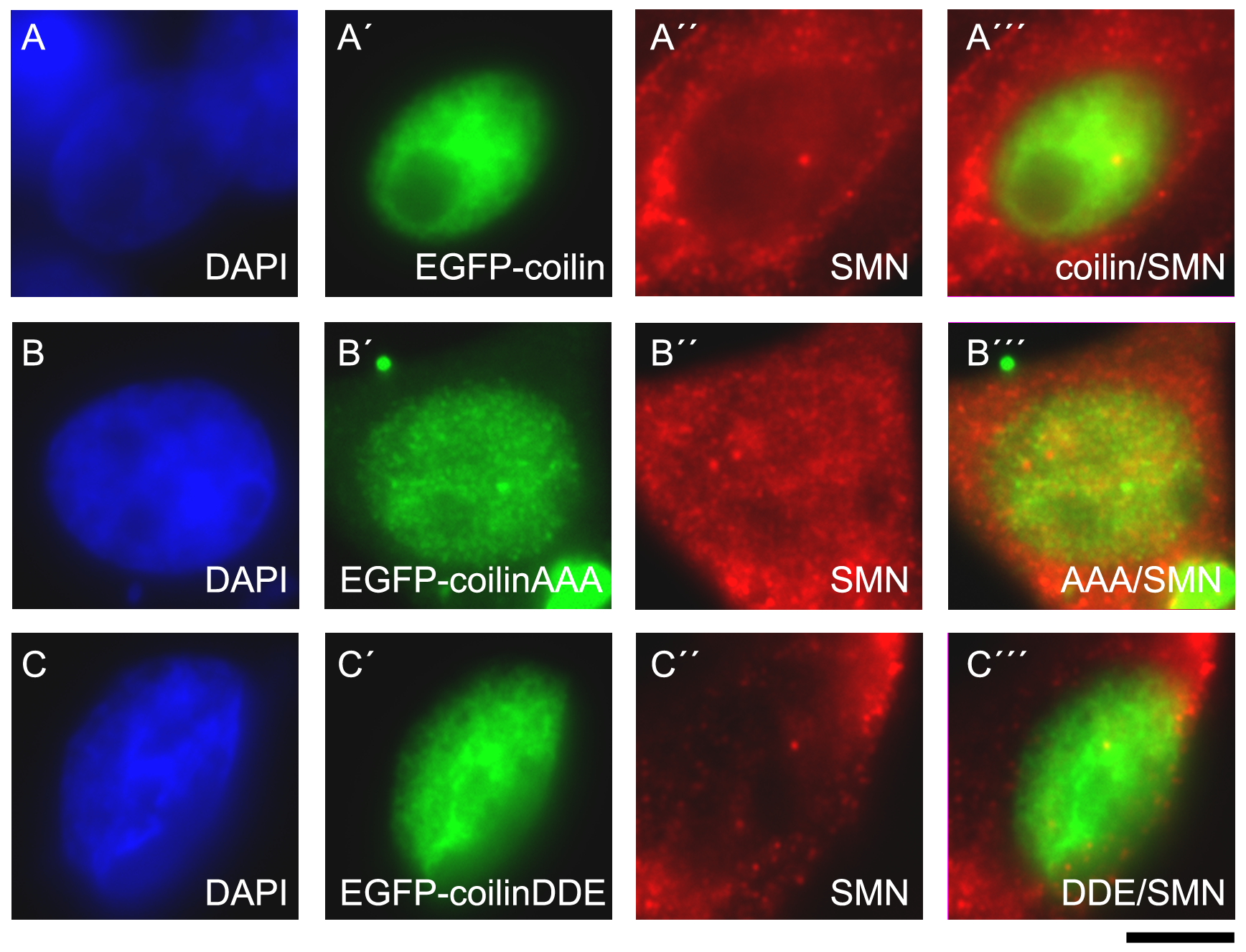

Supplement: Figure S2 — Coilin constructs are similarly distributed in the nucleus of PC12 cells. After transfection with human pEGFP-coilin (A), pEGFP-coilinAAA (B) or pEGFP-coilinDDE (C), respectively, and NGF differentiation for 72 hours, PC12 cells were immunostained for SMN. Overexpressed coilin was not found to be accumulated in Cajal bodies in most of the cells. Most SMN positive nuclear bodies were negative for human coilin, too. Scale bar, 5 µm. (TIF) [file pone.0082871.s002.tif]
